# The survival impact of upfront or sequential chemoradiotherapy in locally advanced esophageal squamous cell carcinoma

**DOI:** 10.3389/fonc.2026.1785745

**Published:** 2026-04-29

**Authors:** Xuechun Luo, Fan Peng, Xi Lin, Yun Li, Zhenyu Ding

**Affiliations:** 1 Department of Biotherapy, Cancer Center, West China Hospital, Sichuan University, Chengdu, Sichuan, China; 2 National Clinical Research Center for Geriatrics, West China Hospital, Sichuan University, Chengdu, Sichuan, China; 3 Department of Thoracic Surgery/Oncology, Mianzhu People’s Hospital (West China Mianzhu Hospital Sichuan University), Mianzhu/Deyang, Sichuan, China; 4 Department of Oncology, Suining Central Hospital, Suining, Sichuan, China

**Keywords:** conversion therapy, definitive chemoradiotherapy (dCRT), esophageal squamous cell carcinoma (ESCC), immune checkpoint inhibitors (ICBs), multicenter analysis, survival analysis

## Abstract

**Background:**

The treatment of locally advanced unresectable esophageal squamous cell carcinoma (ESCC) is a clinical challenge with limited therapeutic consensus. The immune checkpoint inhibitor (ICI)-based conversion therapies emerged as an option besides definitive chemoradiotherapy (DCRT). Especially for those returned to DCRT after unsuccessful conversion therapy (salvage), the clinical outcome remained overlooked when compared with upfront DCRT.

**Methods:**

This study retrospectively analyzed the clinical data of patients with locally advanced ESCC who had received DCRT either as upfront or salvage therapy from 2018 to 2024. Overall survival (OS), event-free survival (EFS), progression-free survival (PFS) and objective remission rate (ORR) were assessed.

**Results:**

Totally 848 patients from 3 medical centers were screened, and 91 patients were included. The overall objective remission rate (ORR) was achieved in 28 cases (68.29%) in the salvage group, higher than the upfront group (26.00%, P < 0.001). Following conversion therapy, 11 patients achieved an ORR of 26.83%. After completing salvage DCRT, CR was observed in 2 patients (4.88%) and PR in 26 patients (63.41%). PFS was 20.3 months (95% CI: 11.6-28.9 months) and 6.4 months in the salvage and upfront group (HR 0.36, 95%CI: 0.19-0.66; P = 0.001). The median OS for the entire cohort was 24.6 months (95% CI 19.5-29.8 months), with a median OS of 42.2 months in the salvage group compared with 15.9 months in the upfront group (HR 0.39, 95% CI: 0.22-0.69; P = 0.001). The salvage group showed significantly longer OS than the upfront group, with 1-year OS rates of 95.1% and 56.9%, and 2-year OS rates of 68.8% and 36.6%, respectively.

**Conclusion:**

The salvage DCRT significantly improved the survival outcomes of patients with locally advanced unresectable ESCC over upfront DCRT. These findings support the clinical feasibility of this strategy and underscore the need for prospective randomized trials to validate its role as a new treatment paradigm.

## 1 Introduction

Esophageal cancer ranks as the seventh leading cause of cancer-related mortality worldwide (1). China bears a disproportionate burden of this disease, accounting for over 50% of global incident cases annually, with more than 90% being squamous cell carcinoma (ESCC) (2). Owing to its insidious onset and the lack of effective early diagnostic biomarkers, approximately half of patients with ESCC are diagnosed at unresectable or metastatic stages (3), for whom survival outcomes remain dismal (4). In particular, patients with clinical T4 disease and/or bulky lymph node involvement often present with suspected invasion of adjacent organs, rendering radical resection technically challenging or infeasible and resulting in an unfavorable prognosis (5). For locally advanced unresectable ESCC, the RTOG 85–01 trial has established definitive chemoradiotherapy (DCRT) as the standard first-line treatment, yet long-term survival remains unsatisfactory (6).

In recent years, immune checkpoint inhibitors (ICIs) have revolutionized the treatment landscape for esophageal cancer. Pivotal trials, such as KEYNOTE-590 and CheckMate-648, have established the survival benefits of pembrolizumab or nivolumab in combination with fluoropyrimidine-platinum chemotherapy, demonstrating a significant extension of overall survival (OS) in patients with advanced disease (7–12). Furthermore, several studies have reported that immunochemotherapy (ICT) can effectively downstage initially unresectable, locally advanced esophageal squamous cell carcinoma (ESCC), enabling successful conversion to surgical candidacy and achieving R0 resection (13–15). Although large-scale Phase III trials are currently lacking, preliminary evidence suggests that combining immunotherapy with chemoradiotherapy holds significant promise for the management of locally advanced ESCC (16–20). These findings collectively indicate that ICI-based regimens possess potent conversion potential, potentially leading to superior long-term survival outcomes.

Against this background, ICI-based conversion therapy has emerged as a potential treatment option for patients with initially unresectable locally advanced ESCC, in addition to the recommended DCRT. However, the optimal treatment strategy remains unclear. Notably, a proportion of patients ultimately proceed to DCRT following failure of conversion therapy. Whether prior exposure to ICI-based conversion therapy adversely affects the efficacy and safety of subsequent DCRT remains an important and unresolved clinical question. In other words, it is unclear whether outcomes differ between patients receiving DCRT as upfront treatment and those undergoing DCRT as salvage therapy after unsuccessful conversion therapy.

This multicenter retrospective study was therefore designed to compare the efficacy and toxicity profiles of these two treatment strategies. Our aim is to provide real-world evidence to inform clinical decision-making and to explore the feasibility of establishing conversion therapy followed by DCRT as a potential alternative treatment paradigm for locally advanced ESCC.

## 2 Methods

### 2.1 Patients

This study included patients who received radical-intent treatment from 3 medical centers (West China Hospital of Sichuan University, Mianzhu People’s Hospital, and Suining Central Hospital) between January 2018 and May 2024. All the patients suffered from histologically confirmed ESCC, with an age between 18-85. The tumor stage was clinically evaluated, either with cT2–4 N0/N + M0 or M1 disease limited to supraclavicular lymph node metastasis. In addition, the enrolled patients had multistation bulky lymphadenopathy, or lymph node involvement in conjunction with other medical comorbidities, which were considered unresectable by the multimodality treatment (MDT) group. Patients diagnosed with esophageal adenocarcinoma or other subtypes, combined with other cancers, with tumor bleeding, esophageal fistula, distant organ metastasis, severe complications, concomitant malignancies, and incomplete medical records were all excluded. All the included patients demonstrated sufficient hematological, liver and kidney functions.

This study complies with the Declaration of Helsinki and has been approved by the Institutional Review Board (No. 2024-2390). Owing to the retrospective nature of the research, the requirement for informed consent has been waived.

### 2.2 Treatments

Conversion regimens consisted of ICIs combined with platinum-based chemotherapy. Chemotherapy regimens included paclitaxel(135-175mg/m^2^ day 1)/albumin-bound paclitaxel (260 mg/m^2^ day 1) plus carboplatin (AUC 4–5 day 1) or cisplatin (25 mg/m2 days 1–3), administered every 3 weeks for 2–4 cycles. PD-1 inhibitors comprised Camrelizumab, Tislelizumab, Sintilimab, Pembrolizumab, Toripalimab and Nivolumab. Radiotherapy employed intensity-modulated radiotherapy (IMRT) or volumetric modulated arc therapy (VMAT) using 6–10 MV photon beams, with daily image guidance via cone-beam computed tomography (CBCT). The definitive radiotherapy group received 50–60 Gy in 25–30 fractions, with 50.4 Gy covering the clinical target volume (CTV) and up to 60 Gy to the planning gross tumor volume (PGTV). Target volume delineation strictly adhered to imaging standards: the gross tumor volume (GTVp) and positive lymph nodes (GTVnd) were confirmed via contrast-enhanced CT. The clinical target volume (CTV) extended 3 cm proximally and distally from the GTVp, plus 0.5–1 cm radially, encompassing the selective lymphatic drainage area. The planned target volume (PTV) was expanded three-dimensionally by 0.5–0.8 cm beyond the CTV to account for respiratory motion and positioning errors.

### 2.3 Tumor evaluation

The treating physicians evaluated the tumor response according to the RECIST 1.1 criteria. Treatment-induced remissions were categorized as complete response (CR), partial response (PR), stable disease (SD), and disease progression (PD). Overall response rate (ORR) encompassed both CR and PR. The overall response rate (ORR) was defined as the proportion of patients achieving either CR or PR. Survival endpoints were strictly defined: overall survival (OS) was measured from the initiation of treatment to death from any cause; event-free survival (EFS) was calculated from the initiation of treatment to the first occurrence of disease progression rendering surgery unfeasible, local or distant recurrence, or death; and progression-free survival (PFS) was defined as the time from treatment initiation to documented disease progression or death from any cause. Toxicity was assessed according to the Common Terminology Criteria for Adverse Events version 5.0 (CTCAE 5.0). Tumors were staged according to the American Joint Committee on Cancer (AJCC) TNM staging system, 8th edition. For patients receiving conversion therapy, efficacy was assessed every two cycles using contrast-enhanced chest/abdominal CT, neck CT, or ultrasound according to RECIST 1.1. Follow-up evaluation was performed 1 month after completion of treatment, then every 3–6 months for the first 2 years and every 6 months for the next 3 years, including history taking, physical examination, laboratory tests, and CT scans. In addition, esophagogastroscopy, bronchoscopy, barium or ultrasonographic esophagography, PET-CT scan and other investigations were scheduled if clinically indicated. The data cutoff occurred on 30 December 2024.

### 2.4 Statistical analyses

Continuous variables were compared using independent-samples t-tests or Mann-Whitney U tests, while categorical variables were analyzed using Chi-square tests or Fisher’s exact tests, as appropriate. The primary endpoint was OS, with secondary endpoints comprising PFS, ORR, and treatment-related toxicities. Survival outcomes were estimated using the Kaplan-Meier method, and between-group differences were assessed via the log-rank test. Prognostic factors associated with PFS and OS were identified through univariate and multivariate Cox proportional hazards regression models. All reported p-values were two-sided, and statistical significance was defined as p < 0.05.

Statistical analyses were performed using SPSS version 27.0 (IBM Corp., Armonk, NY, USA) and R version 4.4.2 (R Foundation for Statistical Computing, Vienna, Austria). Variables demonstrating clinical relevance or those with p < 0.20 in the univariate analysis were incorporated into the initial multivariate Cox regression models. A backward stepwise approach based on the likelihood ratio (LR) was further employed, considering variables with p < 0.05 for final inclusion. The median follow-up duration was estimated using the reverse Kaplan-Meier method. The proportional hazards (PH) assumption for the Cox models was rigorously verified through the examination of Schoenfeld residuals.

## 3 Results

### 3.1 Patient characteristics

**Figure 1:**
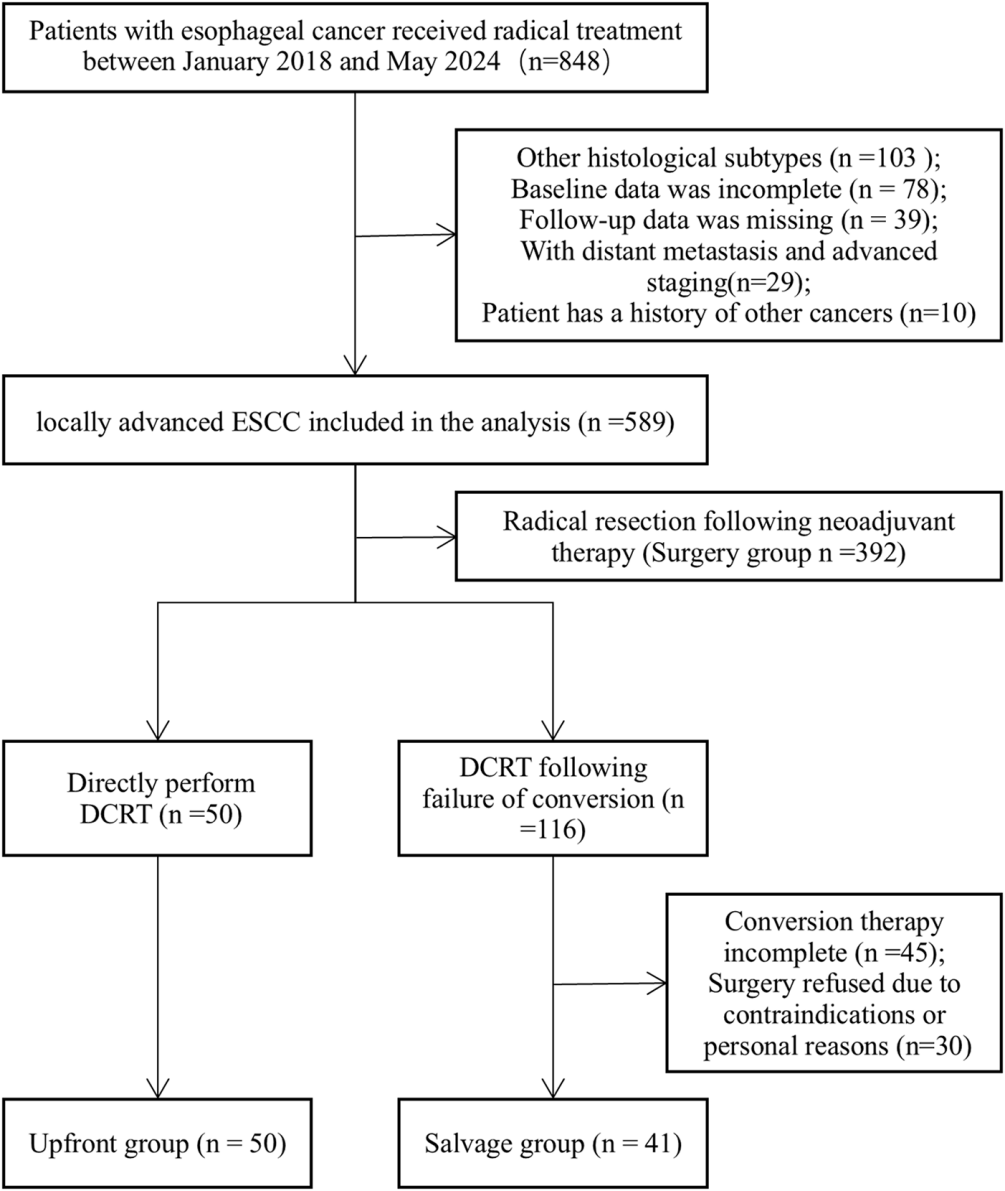
Patient recruitment and inclusion for analysis.

Of the 848 patients initially screened, 91 who received definitive chemoradiotherapy (DCRT) were enrolled in the final analysis (**Figure 1**). Among the study cohort, 50 patients (54.9%) were treated with upfront dCRT (the upfront group), while the remaining 41 patients (45.1%) received dCRT as a salvage treatment following unsuccessful conversion therapy (the salvage group). The baseline characteristics of the participants are summarized in **Table 1**. Patient demographics and baseline characteristics n (%) (n = 91). No significant differences were observed between the two groups regarding gender, age, alcohol consumption, Eastern Cooperative Oncology Group (ECOG) performance status, body mass index (BMI), nutritional risk score (NRS), serum albumin concentration, tumor length, tumor location, or clinical staging (all P > 0.05). The overall cohort was predominantly male (87.9%), with a median age of 67 years (range: 51–84). Notably, a history of smoking was significantly more prevalent in the upfront group (n = 41, 82.0%) compared to the salvage group (n = 20, 48.8%; P = 0.002).

**Table 1:** Patient demographics and baseline characteristics n (%) (n = 91).

Characteristics	Salvage group	Upfront group	P value
	(N = 41)	(N = 50)	
Gender			*0.100*
Male	33 (80.5)	47 (94.0)	
Female	8 (19.5)	3 (6.0)	
Age (years)			*0.140*
< 65	11 (26.8)	22 (44.0)	
≥ 65	30 (73.2)	28 (56.0)	
Smoking status			*0.002*
Yes	20 (48.8)	41 (82.0)	
No	21 (51.2)	9 (18.0)	
Alcohol consumption			*0.072*
Yes	22 (53.7)	37 (74.0)	
No	19 (46.3)	13 (26.0)	
ECOG PS			*1.000*
0	23 (56.1)	27 (54.0)	
1-2	18 (43.9)	23 (46.0)	
BMI (kg/m^2^)			*0.895*
< 18.5	6 (14.6)	7 (14.0)	
18.5-23.9	27 (65.9)	35 (70.0)	
≥ 24	8 (19.5)	8 (16.0)	
Albumin (g/L)	39.7 ± 2.91	39.9 ± 4.46	*0.753*
Tumor length (cm)	5.53 ± 2.08	5.48 ± 2.52	*0.077*
Tumor location			*0.574*
Upper	7 (17.1)	6 (12.0)	
Middle	17 (41.5)	26 (52.0)	
Lower	17 (41.5)	18 (36.0)	
cT stage			*0.607*
T2-3	24 (58.5)	33 (66.0)	
T4	17 (41.5)	17 (34.0)	
cN stage			*0.480*
N0-1	19 (46.3)	28 (56.0)	
N2-3	22 (53.7)	22 (44.0)	
cM stage			*0.307*
M0	40 (97.6)	45 (90.0)	
M1	1 (2.4)	5 (10.0)	
Stage (AJCC 8th)			*0.606*
II-III	19 (46.3)	27 (54.0)	
IV	22 (53.7)	23 (46.0)	

ECOG PS, Eastern Cooperative Oncology Group performance status; BMI: Body Mass Index.

### 3.2 Clinical effects

**Figure 2:**
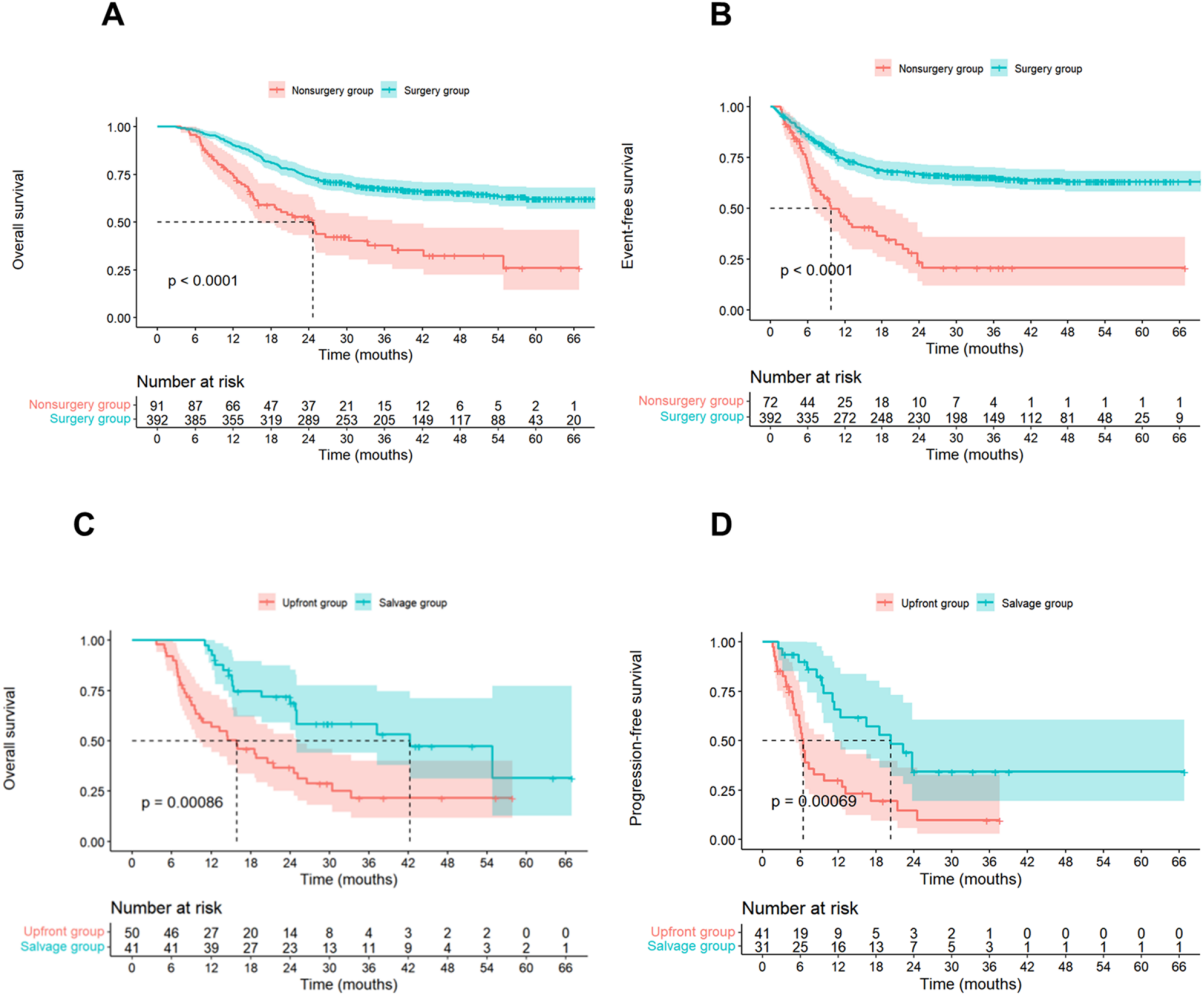
**(A, B)** Event-Free Survival and overall survival for patients with surgery and nonsurgery groups. **(C, D)** Progression-free survival and overall survival comparisons between the salvage group and upfront group.

Initially, we evaluated the clinical efficacy of conversion therapy in patients with initially unresectable, locally advanced ESCC. For those who underwent successful surgical resection following conversion therapy, both median OS and EFS were not reached (NR). These outcomes were significantly superior to those of patients who failed to receive surgery after conversion therapy (median OS: 24.6 months; median EFS: 9.8 months; OS: HR = 0.38, 95% CI: 0.28–0.53, P < 0.001; EFS: HR = 0.34, 95% CI: 0.24–0.48, P < 0.001) (**Figures 2A, B**).

Subsequently, we compared the clinical outcomes of the salvage group (dCRT following unsuccessful conversion) with the upfront group (direct dCRT). Across the entire cohort, the median follow-up duration was 33.3 months (95% CI: 27.9–38.7 months), the median PFS was 9.8 months (95% CI: 5.6–14.0 months), and the median OS was 24.6 months (95% CI: 19.5–29.8 months). The salvage group demonstrated a significantly longer median OS (42.2 months, 95% CI: 20.8–63.7 months) compared to the upfront group (15.9 months, 95% CI: 8.9–23.0 months), with an HR of 0.39 (95% CI: 0.22–0.69; P = 0.001) (**Figure 2C**). The 1-year and 2-year OS rates in the salvage group were 95.1% (95% CI: 88.8%–100.0%) and 68.8% (95% CI: 55.4%–85.3%), respectively, while those in the upfront group were 56.9% (95% CI: 44.5%–72.7%) and 36.6% (95% CI: 25.0%–53.6%). Similarly, the salvage group exhibited a significantly prolonged median PFS (20.3 months, 95% CI: 11.6–28.9 months) relative to the upfront group (6.4 months, 95% CI: 5.7–7.1 months; HR = 0.36, 95% CI: 0.19–0.66; P = 0.001) (**Figure 2D**). The 1-year and 2-year PFS rates followed a similar trend, reaching 65.8% and 34.3% in the salvage group versus 29.9% and 14.6% in the upfront group, respectively.

Tumor response outcomes are summarized in **Table 2**. The salvage group achieved a significantly higher objective response rate (ORR) compared to the upfront group (68.29% vs. 26.00%, P < 0.001). Following initial conversion therapy, CR was achieved in 1 patient (2.44%), PR in 10 patients (24.39%), and SD in 29 patients (70.73%). After completion of salvage dCRT, the response deepened, with CR noted in 2 patients (4.88%) and PR in 26 patients (63.41%).

**Table 2:** Evaluation of efficacy after treatment.

Tumor response	Salvage group		Upfront group
	After conversion therapy, n (%)	After salvage DCRT, n (%)	After directly DCRT, n (%)
CR	1 (2.44)	2 (4.88)	1 (2.00)
PR	10 (24.39)	26 (63.41)	12 (24.00)
SD	29 (70.73)	10 (24.39)	22 (44.00)
PD	1 (2.44)	3 (7.32)	13 (26.00)
NE	0 (0.00)	0 (0.00)	2 (4.00)

**Figure 3:**
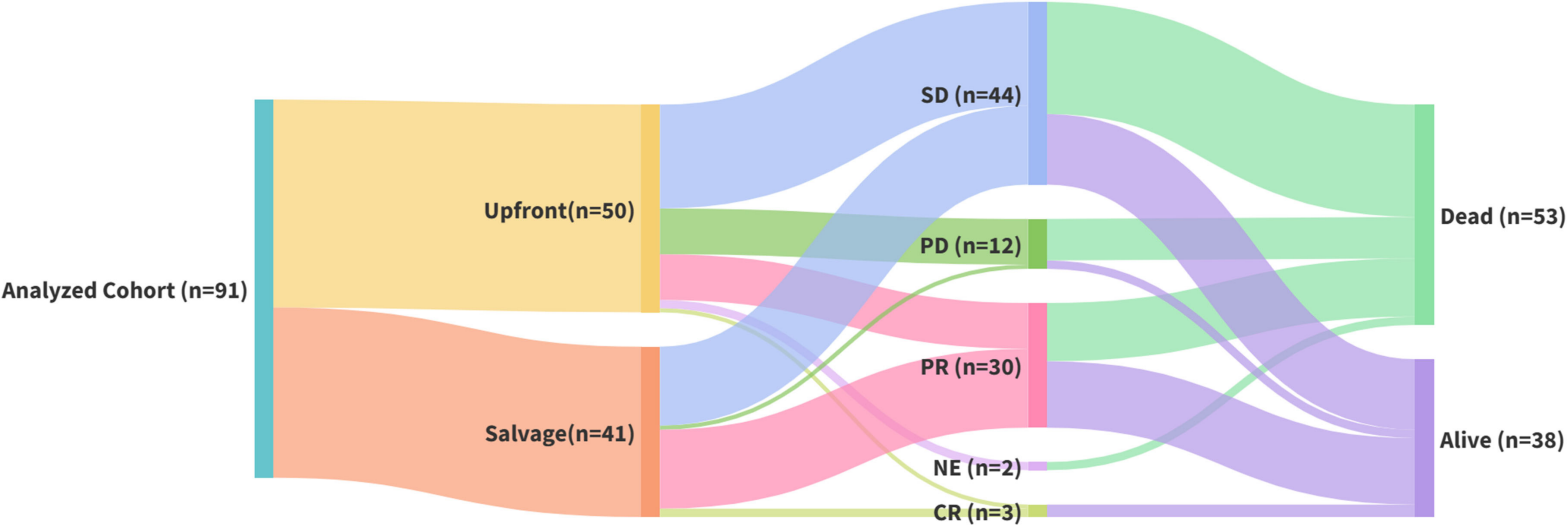
Sankey diagram. The first node represents the 91 patients with esophageal squamous cell carcinoma (ESCC) who were included in the analysis and received radical radiotherapy. The second node shows the subgroups, the upfront group (50 patients), and the salvage group (41 patients). The third node shows patients who were excluded from the analysis for various reasons, and the efficacy evaluations of patients in both groups after completion of all treatments, including complete remission (CR), partial remission (PR), stable disease (SD), disease progression (PD) and non-evaluable (NE). The last node shows patient outcomes, including those who succumbed to the disease (53) and remained alive (38).

**Figure 3** illustrates the clinical status across cohorts; among surviving patients, 23 (60.53%) belonged to the salvage group, whereas 35 of the deceased patients (66.04%) were from the upfront group.

### 3.3 Univariate and multivariate analysis

Prognostic influences were systematically evaluated using univariate and multivariate Cox proportional hazards regression analyses. Within the entire study cohort, multivariate analysis identified smoking history (HR = 2.861, 95% CI: 1.239–6.606; P = 0.014) and AJCC stage (HR = 0.117, 95% CI: 0.032–0.421; P = 0.001) as independent prognostic factors for PFS (**Table 3**). Regarding OS, multivariate Cox regression revealed that both BMI (HR = 0.474, 95% CI: 0.228–0.644; P = 0.046) and the treatment strategy (conversion therapy vs. upfront DCRT: HR = 0.377, 95% CI: 0.212–0.670; P < 0.001) were independently associated with survival outcomes (**Table 4**).

**Table 3:** Prognostic factors of PFS by univariate and multivariate analysis.

Variables	Univariate analysis		Multivariate analysis	
	HR (95%CI)	*P* value	HR (95%CI)	*P* value
Gender (Female vs. Male)	1.734 (0.684-4.399)	*0.246*		
Age (< 65 vs. ≥ 65)	0.629(0.349-1.135)	*0.124*		
Smoking status (No vs. Yes)	3.499(1.684-7.273)	*<0.001*	2.861(1.239-6.606)	*0.014*
Alcohol consumption (No vs. Yes)	2.218(1.169-4.208)	*0.015*		
ECOG PS (0 vs. 1-2)	0.918(0.513-1.643)	*0.772*		
BMI (< 18.5 vs. ≥ 18.5)	0.551(0.245-1.242)	*0.151*		
Albumin (g/L)	1.018(0.940-1.102)	*0.664*		
Tumor length	1.001(0.872-1.150)	*0.983*		
Tumor location (Upper reference)		*0.133*		
Middle	1.012(0.412-2.486)	*0.979*		
Lower	1.840(0.924-3.665)	*0.083*		
cT stage (T2–3 vs. T4)	1.194(0.648-2.200)	*0.569*		
cN stage (N0 vs. N +)	0.920(0.329-2.574)	*0.874*		
cM stage (M0 vs. M1)	1.301(0.510-3.319)	*0.582*		
Clinical stage (II vs. III-IV)	0.211(0.062-0.720)	*0.013*	0.117(0.032-0.421)	*0.001*
Whether conversion therapy was received (No vs. Yes)	0.357(0.193-0.663)	*0.001*	0.542(0.269-1.093)	*0.087*

### 3.4 Treatment-related adverse events

The overall incidence of treatment-related adverse events (TRAEs) was comparable between the upfront and salvage groups (**Table 4**). Notably, the salvage group experienced a lower frequency of hematological toxicities than the upfront group, including leukopenia (41.5% vs. 46.0%), neutropenia (14.6% vs. 22.0%), thrombocytopenia (9.8% vs. 18.0%), and anemia (24.4% vs. 36.0%), although these differences did not reach statistical significance (all P > 0.05). A similar trend was observed for severe (Grade ≥ 3) hematological TRAEs. The incidences of elevated transaminase levels and renal toxicities remained comparable between the two cohorts. Regarding immune-related adverse events (irAEs), the occurrences of skin rash, hypothyroidism, and other toxicities (e.g., peripheral neuropathy and arrhythmia) were relatively low and manageable. Importantly, two treatment-related deaths occurred, both of which were confined to the upfront group: one patient succumbed to sudden cardiac arrest, and the other died of acute respiratory distress syndrome (ARDS) secondary to post-radiotherapy pulmonary injury.

**Table 4:** Treatment-related adverse events.

	Any grade, n (%)			Grade ≥ 3, n (%)		
	Salvage	Upfront	P value	Salvage	Upfront	P value
Leukopenia	17(41.5)	23(46.0)	*0.664*	4(9.8)	7(14.0)	*0.768*
Neutropenia	6(14.6)	11(22.0)	*0.370*	2(4.9)	8(16.0)	*0.177*
Thrombocytopenia	4(9.8)	9(18.0)	*0.263*	1(2.4)	3(6.0)	*0.756*
Anemia	10(24.4)	18(36.0)	*0.233*	1(2.4)	5(10.0)	*0.307*
Elevated alanine aminotransferase	4(9.8)	6(12.0)	*0.997*	1(2.4)	2(4.0)	*1.000*
Elevated aspartate aminotransferase	4(9.8)	5(10.0)	*1.000*	1(2.4)	2(4.0)	*1.000*
Elevated serum creatinine	4(9.8)	3(6.0)	*0.697*	0(0.0)	0(0.0)	*-*
Gastrointestinal reactions	6(14.6)	12(24.0)	*0.264*	2(4.9)	3(6.0)	*1.000*
Abnormal myocardial markers	2(4.9)	4(8.0)	*0.687*	1(2.4)	2(4.0)	*1.000*
Radiation esophagitis	3(7.3)	3(6.0)	*1.000*	0(0.0)	1(2.0)	*1.000*
Radiation pneumonitis	0(0.0)	4(8.0)	*0.181*	0(0.0)	3(6.0)	*0.315*
Rash	0(0.0)	2(4.0)	*0.499*	0(0.0)	0(0.0)	*-*
Hypothyroidism	3(7.3)	2(4.0)	*1.000*	0(0.0)	0(0.0)	*-*
Peripheral nerve lesions	2(4.9)	1(2.0)	*0.587*	0(0.0)	0(0.0)	*-*
Arrhythmia	2(4.9)	3(6.0)	*1.000*	1(2.4)	2(4.0)	*1.000*
Canker sore	2(4.9)	3(6.0)	*1.000*	0(0.0)	0(0.0)	*-*
Lower limb venous thrombosis	1(2.4)	2(4.0)	*1.000*	0(0.0)	1(2.0)	*1.000*

## 4 Discussion

This study demonstrates that, in locally advanced ESCC, compared to upfront DCRT, the salvage DCRT following unsuccessful conversion therapy yields superior clinical efficacy. The salvage approach achieved a significantly higher ORR and remarkably prolonged both OS and PFS. Specifically, the median OS in the salvage group reached 42.2 months, with 1-year and 2-year OS rates of 95.1% and 68.8%, respectively. Furthermore, the median PFS was 20.3 months, with 1-year and 2-year PFS rates of 65.8% and 34.3%. Collectively, these findings indicate a pronounced survival benefit of the salvage strategy over upfront DCRT, suggesting that even when conversion to surgery is not feasible, initial ICI-based induction therapy may still prime the tumor for improved outcomes during subsequent radiotherapy.

Following the landmark success of immune checkpoint inhibitors (ICIs) in malignancies such as melanoma and lung cancer (21, 22), their therapeutic indications have rapidly expanded to include esophageal cancer (23). Accumulating evidence from multiple clinical trials on combined immunochemotherapy (ICT) has demonstrated a robust increase in objective response rates (ORR), reaching up to 70% in select cohorts (8, 9, 11, 24, 25). Given this high radiological response, ICI-containing conversion regimens have significantly enhanced the feasibility of surgical conversion for initially unresectable cases. For instance, Huang et al. underscored that such induction therapy markedly increases the probability of successful surgical transition. Furthermore, real-world data have confirmed that the surgical conversion rate for ICT can reach 74.8%, with an R0 resection rate as high as 94% (13)— a finding that is highly consistent with the outcomes observed in the surgical cohort of the present study.

The landmark RTOG 85–01 trial established definitive chemoradiotherapy (dCRT) as the standard of care for initially unresectable, locally advanced ESCC. Nevertheless, long-term survival remains suboptimal, with a plateaued five-year survival rate of merely 26% (6). In the decades following this trial, no substantial survival breakthroughs have been achieved through conventional strategies. In contrast, conversion therapy facilitates significant tumor shrinkage and downstaging, thereby creating previously unavailable surgical opportunities. Data from our present study indicate a robust surgical conversion rate of 82.9%, underscoring that induction immunochemotherapy significantly enhances resectability and serves as a critical pathway toward achieving curative outcomes.

However, patients who do not meet the criteria for surgical intervention following conversion therapy are typically referred back to dCRT. In our study, for this subset of patients (45.1%), subsequent salvage dCRT still yielded substantial clinical benefits: the ORR reached 68.29%, which was markedly higher than the 26.0% observed in the upfront dCRT group, ultimately leading to prolonged PFS and OS. These findings are supported by the long-term follow-up of the JCOG1510 study, which indicated that even among patients failing conversion therapy, the two-year OS rate following salvage treatment reached 41.2%—a 12.7% improvement over historical controls (18). Furthermore, prior conversion therapy has been reported to substantially elevate the complete response (CR) rate of subsequent dCRT to 62%, with a corresponding one-year OS rate of 78.4% (26). In our cohort, the conversion strategy also markedly improved the 2-year EFS rate, demonstrating a 27.5% absolute increase compared to the upfront group (13), further validating the therapeutic synergy between induction immunochemotherapy and definitive radiotherapy.

Therefore, compared with upfront dCRT, ICI-based conversion therapy not only offers superior therapeutic feasibility but also delivers significant survival benefits for patients with locally advanced ESCC (8, 27–29). Our study underscores that conversion therapy not only facilitates surgical opportunities for approximately half of the cohort but, perhaps more critically, confers a survival advantage through robust systemic disease control and potential radiosensitization. These findings provide compelling real-world evidence to support the integration of ICT as a standard-of-care conversion strategy for initially unresectable, locally advanced ESCC.

The underlying mechanism for this therapeutic synergy resides in the dual-level enhancement—both local and systemic—exerted by conversion therapy. Locally, induction immunotherapy orchestrates a favorable shift in the tumor microenvironment (TME) by promoting CD8^+^ T-cell infiltration, facilitating antigen release, and elevating the levels of proinflammatory cytokines such as IFN-γ and TNF, which collectively prime the tumor for enhanced radiosensitivity (30–38). Systemically, ICIs activate a robust anti-tumor immune response that persistently eliminates circulating tumor cells (CTCs) and occult micrometastases, thereby substantially mitigating the risks of locoregional recurrence (LRR) and distant metastasis (DM) (39–43). This is reflected in our findings, where the 2-year local control rate reached 78%, representing a notable improvement over the 62% reported for conventional regimens (41, 44–47).

Moreover, ICI-based conversion therapy demonstrated a favorable safety profile in our study. While no statistically significant differences in the overall incidence of TRAEs were observed between the two cohorts, the salvage group exhibited a lower frequency of anemia and grade ≥3 adverse events compared to the upfront group. Consistent with our results, pivotal trials such as KEYNOTE-590 and ESCORT-1st (8, 9), have confirmed that the incidence of severe (grade ≥3) toxicities with ICT remains comparable to that of chemotherapy alone (P > 0.05). Notably, the absence of treatment-related deaths in the salvage group further underscores the clinical safety of this sequential approach. Although common radiation-related toxicities, such as radiation pneumonitis, were similar between groups, the salvage group demonstrated superior overall tolerance, particularly regarding the prevention of severe adverse events. These findings align with broader real-world evidence suggesting that prior conversion therapy does not exacerbate radiation-induced injury—with comparable rates of radiation pneumonitis and esophagitis—and may even mitigate the occurrence of high-grade complications (16, 18–20, 48–50).

Several limitations of the present study warrant acknowledgment. Firstly, despite its multicenter retrospective design, the relatively modest sample size may introduce inherent selection bias and potential confounding factors; thus, our findings require further validation in larger, prospective cohorts. Secondly, the heterogeneity in the selection of specific immunotherapy and chemotherapy agents across different centers may have introduced variability in efficacy assessments. Thirdly, the overall objective response rate (ORR) observed suggests that induction regimens could be further optimized to maximize therapeutic benefit. Moreover, the lack of comprehensive biomarker data, such as PD-L1 expression levels and tumor mutational burden, precluded more granular subgroup analyses regarding treatment sensitivity. Additionally, while multivariate adjustments were performed, baseline imbalances between the study groups may have introduced residual confounding that cannot be entirely eliminated. Consequently, these results should be interpreted with appropriate caution. Given these limitations, this study can only provide preliminary, real-world evidence on the efficacy and safety of ICI-based conversion therapy for locally advanced ESCC. These findings are primarily hypothesis-generating and require validation in future prospective studies.

## 5 Conclusion

In this retrospective study, we demonstrated that salvage DCRT significantly improved the survival outcomes of patients with locally advanced unresectable ESCC without a significant toxic burden. These results warrant further validation through prospective randomized controlled trials to establish this strategy as a potential new treatment paradigm in the management of locally advanced ESCC.

## Data Availability

The raw data supporting the conclusions of this article will be made available by the authors, without undue reservation.
